# Magnetic and microscopic investigation of airborne iron oxide nanoparticles in the London Underground

**DOI:** 10.1038/s41598-022-24679-4

**Published:** 2022-12-15

**Authors:** H. A. Sheikh, P. Y. Tung, E. Ringe, R. J. Harrison

**Affiliations:** 1 Department of Earth Sciences, University of Cambridge, Cambridge, CB2 3EQ UK; 2 Department of Materials Sciences, University of Cambridge, Cambridge, CB3 0FS UK

**Keywords:** Environmental impact, Environmental sciences, Environmental monitoring

## Abstract

Particulate matter (PM) concentration levels in the London Underground (LU) are higher than London background levels and beyond World Health Organization (WHO) defined limits. Wheel, track, and brake abrasion are the primary sources of particulate matter, producing predominantly Fe-rich particles that make the LU microenvironment particularly well suited to study using environmental magnetism. Here we combine magnetic properties, high-resolution electron microscopy, and electron tomography to characterize the structure, chemistry, and morphometric properties of LU particles in three dimensions with nanoscale resolution. Our findings show that LU PM is dominated by 5–500 nm particles of maghemite, occurring as 0.1–2 μm aggregated clusters, skewing the size-fractioned concentration of PM artificially to larger sizes when measured with traditional monitors. Magnetic properties are largely independent of the PM filter size (PM_10_, PM_4_, and PM_2.5_), and demonstrate the presence of superparamagnetic (< 30 nm), single-domain (30–70 nm), and vortex/pseudo-single domain (70–700 nm) signals only (i.e., no multi-domain particles > 1 µm). The oxidized nature of the particles suggests that PM exposure in the LU is dominated by resuspension of aged dust particles relative to freshly abraded, metallic particles from the wheel/track/brake system, suggesting that periodic removal of accumulated dust from underground tunnels might provide a cost-effective strategy for reducing exposure. The abundance of ultrafine particles identified here could have particularly adverse health impacts as their smaller size makes it possible to pass from lungs to the blood stream. Magnetic methods are shown to provide an accurate assessment of ultrafine PM characteristics, providing a robust route to monitoring, and potentially mitigating this hazard.

## Introduction

The London Underground (LU) is a popular transport choice for Londoners and visitors, carrying 2 million passengers per day. The concentration of particulate matter (PM_10_, PM_2.5_ and, PM_1_) in the LU is found to be significantly higher than London background PM levels. A previous air pollution monitoring study on the LU suggested that higher PM levels might be associated with the age and depth of the platforms, and the poor ventilation systems^1^. PM_2.5_ concentrations in subway systems similar to the LU have been reported to have concentrations exceeding WHO air quality guidelines for PM. However, the PM level in the LU has attracted less attention. Although some lines of the LU are above the surface, the transport system is considered an indoor environment for which Department for Environment, Food & Rural Affairs (DEFRA UK) has no guideline limits for PM. Previous studies have reported chemical compositions of PM_2.5_ in the LU to be dominantly Fe-oxide (47–67%), 1–2% quartz, other heavy metals, 18% carbon (elemental carbon and organic carbon) and 14% metallic and mineral oxides^1,2^. The sources of Fe-rich PM in the LU originate from different components of the wheel-track-brake system. Previous studies in London^1–3^ and Seoul^4^ have identified that Fe-rich PM is generated by wear of steel components and rails due to friction, wear of train parts such as collector shoes, which are made of cast iron, and Fe-containing brake blocks. Currently in the LU, localised emission vacuums (LEVs) capture a proportion of the welding fumes that are generated when metals are heated above their melting point, vaporise, and condense into aerosols. Therefore, most Fe-rich particles are likely to come from abrasion of wheel-track-brake system (although all except Bakerloo and Piccadilly lines have regenerative braking).

Exposure to ambient air pollution ultrafine particles has been linked to health risks associated with asthma, brain damage^5^, dementia^6^, lung cancer, cardiovascular diseases, reduced cognitive ability^7^. In particular, human health effects of magnetite PM has been linked to Alzheimer’s^8^ and magnetite nanoparticles have also been found in the brain, which could have serious implications^9,10^. However, there have been limited, inconclusive health studies done on the potential health impacts of underground train systems^11,12^ which are rich in Fe-oxide PM. There is, as yet, no definitive evidence that exposure to particles in an underground rail environment is more dangerous than ambient air pollution. In addition, it was previously argued that the underground rail environment is unlikely to pose a health risk to workers and commuters due to the different health effects of Fe-oxides and combustion-generated particles and safer PM concentrations below the recommended workplace standards^2^. Previous toxicology studies done in the Stockholm subway (Fe-rich microenvironment) did not see an increased risk of myocardial infarction in subway drivers compared to other manual workers in Stockholm^13^. However, a recent in vitro study by^14^ using PM from the Bakerloo and Jubilee lines of Baker Street station in the LU did find evidence of increased risk of pneumococcal infection and mortality. Another in vitro study of PM_10_ from the Stockholm subway also found that the air was 40–80 times more genotoxic and 20–40 more potent at causing oxidative stress when compared with an urban street environment^15^. Similarly, an underground railway pollution study found that PM_2.5_ and PM_1.8_ have greater ability to produce reactive oxygen species (ROS) than coarser PM_10_; these particles can penetrate the mucous layer, causing an antioxidant response^16^. It is already known that no level of PM exposure can be considered as a safe health limit^17^ and poor ventilation on platforms and tunnels means that commuters are exposed to high levels of particulates during their journeys.

The mechanisms behind health risks posed by Fe-bearing particles derived from vehicular and industrial sources versus those derived from underground sources are poorly understood and are likely to be a function of several factors (i.e., particle number concentration, morphometrics, surface area, reactivity, and mineralogy/oxidation state). This study aims to provide the most detailed characterisation to date of these properties for London Underground PM. We use a combination of room-temperature, low-temperature and high-temperature magnetic methods, first-order reversal curves (FORCs), scanning electron microscopy (SEM), energy-dispersive X-ray spectroscopy (EDS), transmission electron microscopy (TEM), and 3D electron tomography to distinguish individual particles deposited on PM_10_, PM_4,_ and PM_2.5_ air monitoring filters from ticket halls, platforms, and train operator cabins. In this study, we aim to identify magnetically any systematic differences between Fe-bearing particles (which constitute 50% of the entire PM fraction) at different LU localities, thereby improving our understanding of their mineralogy and particle size distribution.

## Results

### Magnetic characterisation of PM

To determine the mineralogical nature of the Fe-oxide phase in LU PM, we used a combination of a low-temperature (LT) magnetisation protocol^18^ and high-temperature (HT) magnetic susceptibility measurements (see Supplementary Text S1 for details). Our LT magnetization curves (Supplementary Fig. S2) did not show any evidence of a Verwey transition associated with magnetite (usually observed as a loss in remanence at temperatures 80–125 K upon warming from 10 K) or a Morin transition associated with hematite (usually observed as a loss of remanence below 260 K on cooling from room-temperature). HT measurements showed a complete irreversible loss of magnetic susceptibility on heating between 206 and 460 °C (Supplementary Fig. S4), characteristic of maghemite—the fully oxidised, metastable form of magnetite that transforms to hematite irreversibly on heating above 200 °C^19^.

We quantified the total magnetic content in our dust samples using LT saturation isothermal magnetisation remanence (SIRM) measurements at 10 K. LT-SIRM represents the total ferrimagnetic contribution of particles, including the superparamagnetic (SP) particles (diameters <  ~ 30 nm) that are magnetically unstable at room temperature. We observe that 60–77% of the remanence at 10 K is carried particles that are < 30 nm, and that the magnetic contribution from these particles varies between the different air filter samples. Frequency-dependent susceptibility (λ_FD_ %) measurements confirm the abundance of particles near the ∼30 nm threshold (see Supplementary Text S1, Fig. S3).

Room-temperature magnetic analysis identifies the contribution of ferrimagnetic grains that carry stable remanence i.e., anything at or above the single-domain (SD) threshold (i.e., particles ≥ 30–70 nm in diameter). The results suggest the presence of particles with apparent sizes in the range 1–7 µm (Supplementary Text S1, Fig. S1). Although this methodology has been used as an indicator for grain size variation for magnetite samples with uniform grain sizes^20,21^, results presented here should be treated with caution as our samples are dominated by maghemite rather than magnetite, have a non-uniform size distribution of particles, and the magnetic properties are likely to be affected by magnetostatic interactions between particles in clusters.

**Figure 1:**
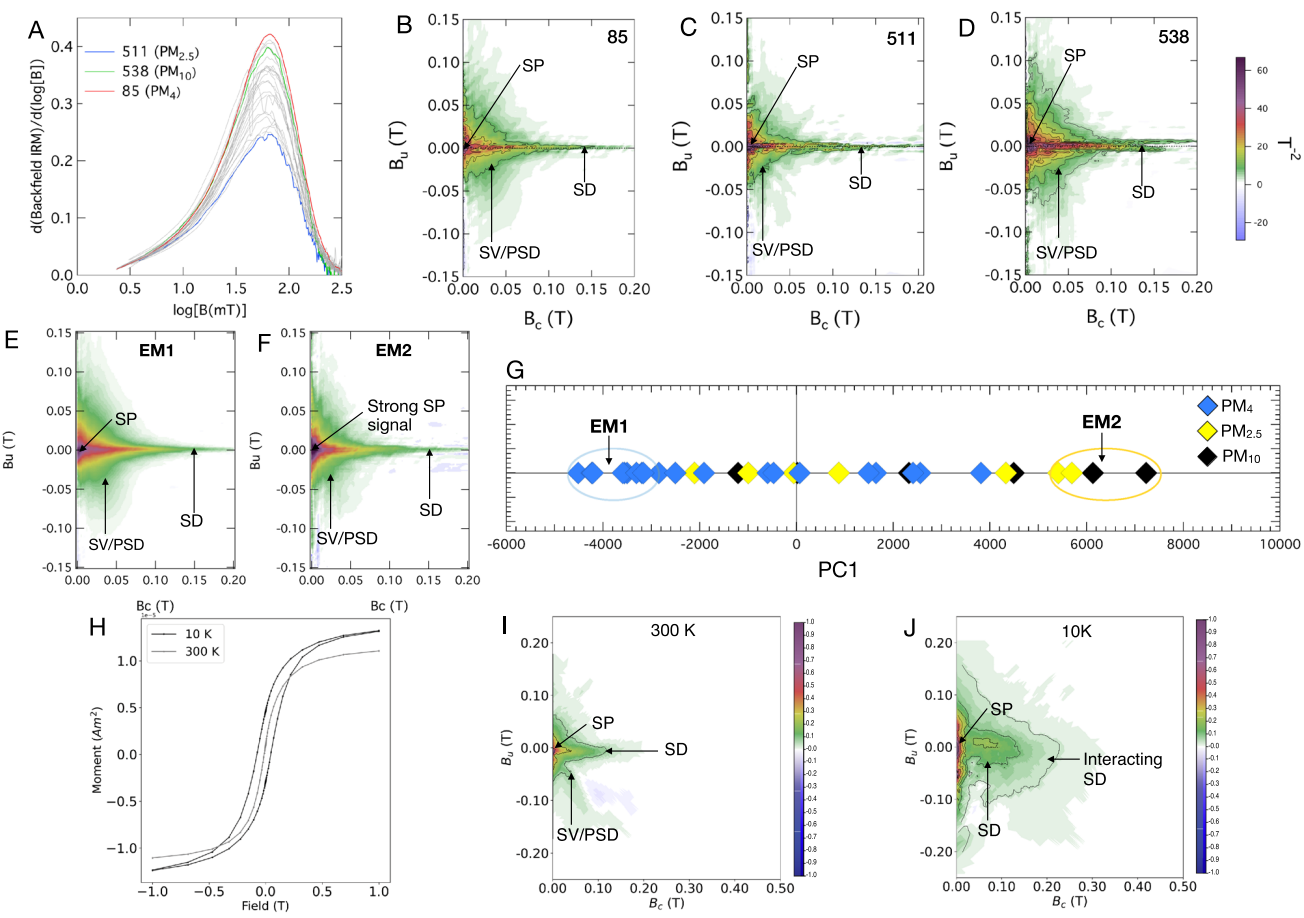
FORCs of representative different size-fractionated filters from the platform, and operator cabins. (**A**) shows log backfield remanent coercivity distribution of all air filter samples analysed (**B**–**D**) all samples show a broadly spread pattern that is indicative of SV/PSD state, and a SD low coercivity ridge. All samples were first normalised to a saturation magnetization (*M*_s_) value of 1. (**B**) also has a high coercivity (HC) ridge extending up to 200 mT. (**E**,**F**) shows the two members (EMs) extracted from FORC-PCA analysis^24^. (**G**) FORC-PCA score plot for mixing space between EM1, and EM2; diamonds are individual FORCs for each sample. (**H**) Low temperature hysteresis curve for sample 511 shows increases in remanence at 10 K vs 300 K. (**I**) Room temperature FORC measured on the MPMS, with coercivity ridge extending to about 150 mT (**J**) Low temperature FORC shows an increase in coercivity to 300 mT at 10 K.

First-order reversal curves (FORC) are a more useful diagnostic tool to determine the range of magnetic domain states (and thereby the range of particle sizes) present, as well as any magnetostatic interactions between particles. We provide a detailed characterisation of London Underground PM dust signatures by comparing high-resolution FORCs at different localities within the LU (Fig. 1B–D) and for different PM size fractions (PM_10_, PM_4,_ and PM_1_). The magnetic signature of our FORCs varies very subtly, driven primarily by variations in very fine SP (< 30 nm) content. To verify the uniformity in our FORC fingerprints, we performed FORC Principal Component Analysis (FORC-PCA) on our processed FORCs (Fig. 1G). All our PM samples lie between two identified endmembers (EM), which contain broadly similar features expressed to subtlety varying degrees. The magnetic signature of EM1, which primarily, but not exclusively, encompasses PM_4_ filter samples from the platform and ticket halls, exhibits: (1) an SD central ridge (particles between 30 and 70 nm) at B_u_ = 0 extending to > 200 mT; (2) a clear vortex/pseudo-single domain (V/PSD) component (particles diameter between 70 and 700 nm); and (3) a vertically asymmetric signal at the origin that is consistent with the presence of SP particles < 30 nm). Similar features are observed in EM2 (consisting primarily, but not exclusively, of PM_2.5_ and PM_10_ air filters from train operator cabins) but with relative greater intensity for the SP component and weaker intensity for the SD and V/PSD and signals compared to EM1. Our remanence FORCs (remFORCs) measured using the irregular measurement algorithm devised by^22,23^ highlights both the SP, SD and V/PSD contributions. Here, the SP signal is isolated in the remFORC diagram in a region that is sensitive to viscous magnetization processes^23^ (see Supplementary Fig. S5).The idea that small variations in proportion of SP particles dictates the subtlety different FORC fingerprints is also confirmed by the uniform shape of the coercivity distribution for all air filter samples, with SP content influencing the height (but not the shape) of the coercivity distribution peak (Fig. 1A). The increase in coercivity from 200 mT (Fig. 1H) to 250 mT observed in the 10 K FORC diagram (Fig. 1J) is caused by the conversion of larger SP particles to stable SD particles on cooling. Low-temperature hysteresis and FORCs indicate the persistence of SP signatures at 10 K, indicating the presence of very small particles (< < 30 nm).

### High-resolution electron microscopy

**Figure 2:**
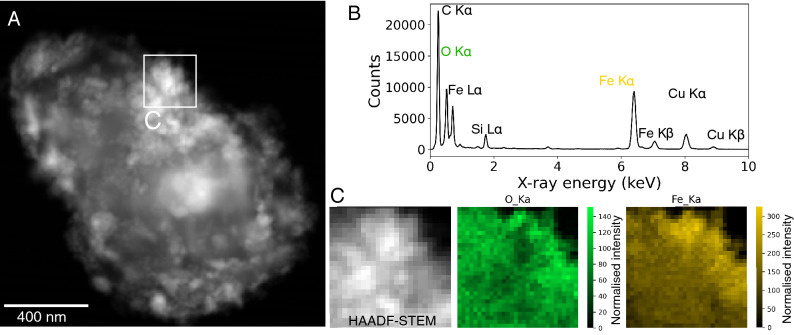
Morphological and chemical fingerprints of representative Fe-bearing particles from LU (**A**) HAADF-STEM imaging of Fe–O nanoparticles (**B**) EDS spectrum from Area C indicates signals from Fe and O, consistent with particles examined using SEM–EDS (see Supplementary Figs. S6–S8). (**C**) Elemental maps for Fe and O from the area of interest highlighted in A.

**Figure 3:**
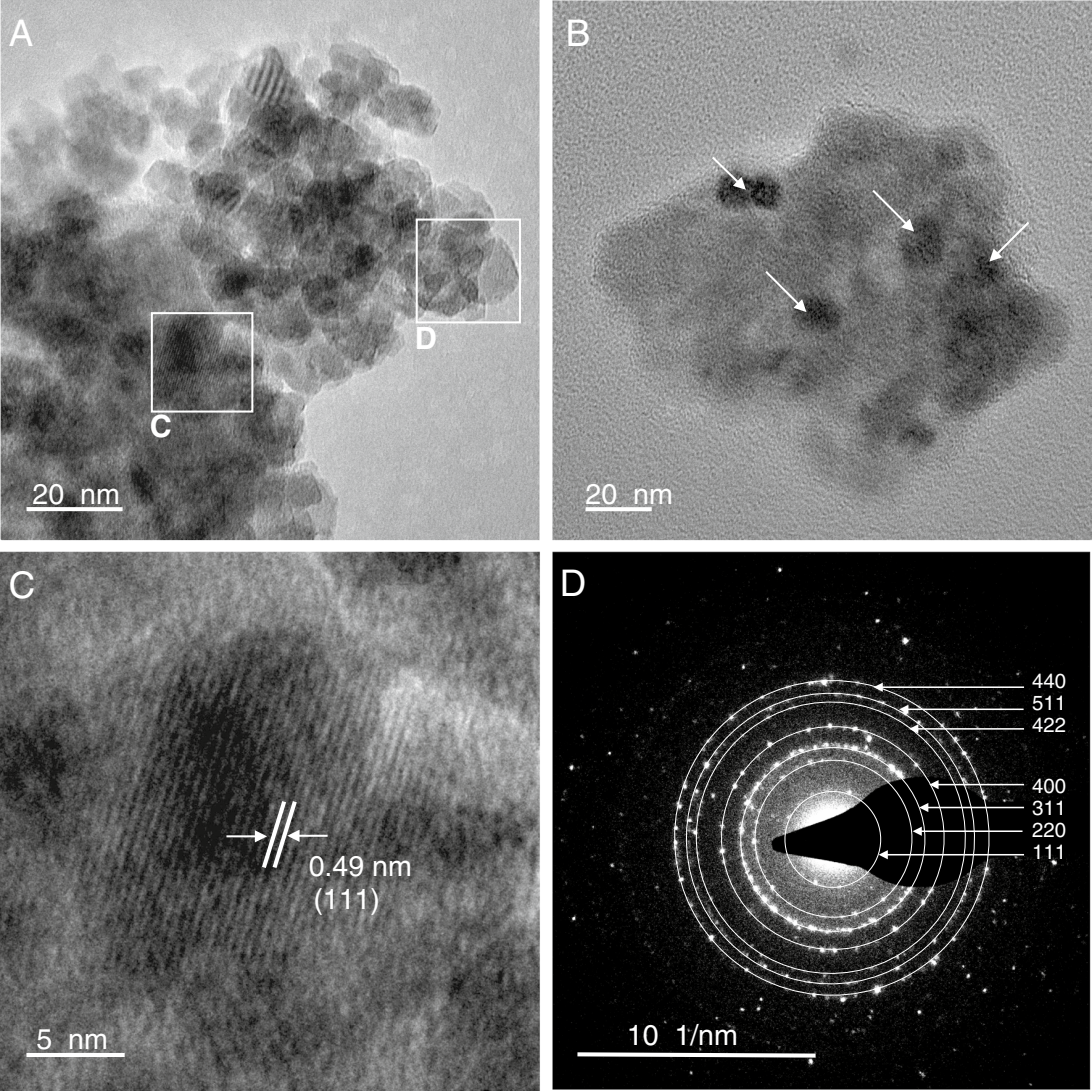
TEM analysis of London Underground sample 180487-86: (**A**) HRTEM image showing aggregates of magnetic nanoparticles, (**B**) darker particles represent individual particles oriented to give strong diffraction contrast (**C**) high- magnification image showing d-spacing for 111 planes of magnetite, (**D**) the selected area diffraction (SAED) pattern from region d shows randomly oriented magnetite nanocrystals.

Initial SEM and EDS analyses of air filter sample 180487-86 (Oxford Circus, Central line E/B, PM_4_ filter) shows agglomerated Fe-bearing particles in micron to sub-micron sized clusters (see Supplementary Fig. S6). The majority of these are dense clusters of nanoparticles as observed in Supplementary Figs. S7 and S8. EDS maps from high-angle annular dark-field (HAADF) imaging in scanning transmission electron microscopy (STEM) mode confirmed the presence of rounded Fe-oxide nanoparticles (Fig. 2 and Supplementary Fig. S10). Electron diffraction patterns of particles from a high-magnification region in Fig. 3A are consistent with a magnetite-maghemite spinel phase (Fig. 3D).

Most particles observed show rounded morphologies (Fig. 3, Supplementary Figs. S7–S10). HRTEM imaging shows particles with dimensions ranging from around 2–30 nm. Some of these agglomerated nanoparticles formed large clusters ranging from 50 nm to 2 μm (Fig. 3A, Supplementary Figs. S7–S10). TEM images analysed show that majority of Fe nanoparticles are observed as clusters, but individual nanocrystals (∼20 nm) are also observed (see Supplementary Fig. S9). To confirm our FORC data reliability, we performed quantification of particle sizes (see Supplementary Table S1) observed by TEM imaging. An aspect ratio (width/length) ranging from 0.5 to 1.00 may be critical in explaining the high coercivity part of the SD ridge in the FORCs (Fig. 1). However, the an aspect ratio of 0.92 confirms the prevalent rounded nature of these particles (see Supplementary Fig. S11).

### Electron tomography

**Figure 4:**
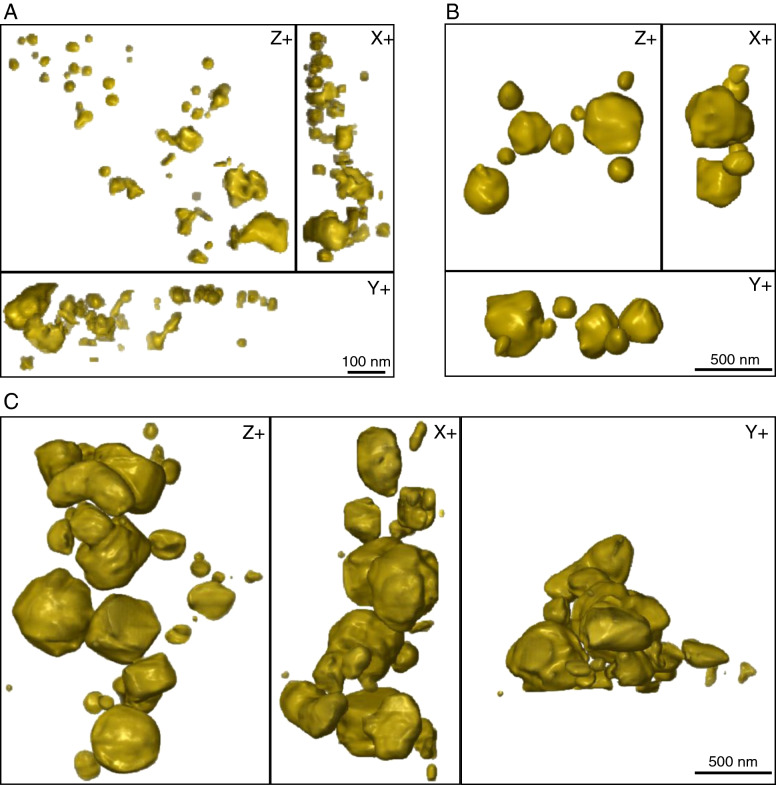
(**A**) Tomographic reconstruction of Fe-bearing particles from 180,487–86, viewed in three different planes: X, Y and Z. (**B**) Tomographic reconstruction of a different region showing relatively finer grain size fractions. (**C**) Rounded particles showing a range of different sizes.

Electron tomography allows us to further investigate the 3D structure and size distribution of nanoparticles. Images of isolated and aggregated particles (Fig. 4) confirm the dominant spherical morphology of particles with few elongated particles (aspect ratio ranging from 0.30 to 0.99), which is in accordance with rounded morphologies observed by SEM, STEM, and HRTEM. The size distribution of particles (see Supplementary Fig. S12) covers the magnetic domain spectrum from SP to V/PSD, consistent with the FORC data (Fig. 1).

## Discussion

Previous studies in subways have used different analytical methods such as electron probe X-ray microanalysis^25^ or SEM/EDS, and XRD^26^ to characterise airborne PM in a subway environment. A study in Seoul Underground used a permanent magnet to separate and quantify magnetic and non-magnetic PM, claiming that the majority of airborne magnetic PM and floor dust was metallic-Fe based on hysteresis curves acquired on a Vibrating Sample Magnetometer (VSM)^26^. They interpret their data to be consistent with metallic Fe based on magnetisation curve data. We did not see evidence of metallic Fe here, suggesting that our particles are dominated by aged particles or resuspended PM and not freshly generated PM. Using low-, high- and room-temperature magnetic measurements, coupled with high-resolution microscopy techniques, our study confirms the abundant presence of SP-SD maghemite particles. FC-ZFC remanence warming curves and RT-SIRM cooling curves (Fig. S2) do not show any evidence of Verwey transition or dampened transition^27^, which would be characteristic of magnetite or surface-oxidised magnetite respectively^28^. Diffraction patterns from TEM (Fig. 3D) and the absence of an obvious Verwey or Morin transition in low temperature-SIRM (LT-SIRM) graphs (Fig. S2) confirm that the Fe-bearing nanoparticles in the LU consist primarily of oxidized, metastable maghemite. Pure maghemite shows perfectly reversible RT-SIRM warming and cooling curves^34^, however, in our case the warming and cooling curves diverge (Fig. S2A,C,E), suggesting conversion to maghemite may not be complete or uniform throughout the samples. A study of PM_10_ samples in the Barcelona metro interpreted metallic flake-like particles to be generated by mechanical wear of lateral brake pads and wheels, followed by oxidation of metallic-Fe to magnetite and maghemite or hematite^29^. Overall, our results allow us to propose that the primary source of particles on PM air filters is from resuspension of Fe-bearing PM upon train arrival at a platform, since only the older particles will have had time to oxidise due to poor ventilation in the microenvironment.

The similarity of all room temperature FORC fingerprints, which does not vary systematically as a function of the conventional PM size fraction (PM_2.5_, PM_4_, and PM_10_) suggests that the magnetic fraction of PM in the LU is the result of a ubiquitous source of ultra-fine particles that are clumped to varying degrees. The similarity of remanent coercivity distributions (Fig. 1A) and the FORC-PCA endmembers (Fig. 1E,F) suggests the primary mode of variation from sample to sample is the relative proportion of the finest (SP) fraction relative to the coarser (SD/V/PSD) fraction. Air filters from different localities produce only slightly differing mixtures of SP, SD, V/PSD signatures at room temperature. At 10 K, there is both a horizontal and vertical broadening of the SD signal, which we interpret as an increase in the fraction of interacting SD particles at low temperature as SP particles in clusters become thermally blocked. The tail of the coercivity distribution, which extends to 300 mT (Fig. 1A) can be explained by either (a) the presence of elongated SD (oxidised) magnetite with an aspect ratio > 0.3, as seen (albeit rarely) in our TEM and tomography particle size data (see Supplementary Figs. S11 and S12) or (b) the nucleation and annihilation of vortex states in metallic Fe nanoparticles (ranging from 32 nm to around 500 nm)^30^. The peak of the backfield coercivity distribution for all our air filter samples lie at around 65 mT (Fig. 1A), similar to coercivity values for vehicular brake residue samples and higher than vehicular exhaust emissions from a previous study^31^. No direct evidence of metallic Fe was found in our microscopy data. FORC signatures of brake-residue samples dominated by metallic-Fe nanoparticles typically show a bi-modal FORC signature of high-coercivity ridge and low-coercivity wings and lack the clear magnetite-like V/PSD signals observed in Lahore^30^. In fact, they look more similar to FORC leaf fingerprints from both Lahore and leaf and lichen FORC fingerprints observed in Rome^32^—likely to be because Fe-rich particles are dominated by brake wear PM and are oxidized over time. Although we cannot rule out some contribution to the high-coercivity signal from metallic Fe, the magnetic features observed here do not require metallic Fe to be present and are consistent with maghemite particles with the sizes, and shapes observed in the microscopy/tomography data, which span the SP, SD to V size range, and have the requisite aspect ratios to create high coercivity signals (see Supplementary Fig. S12). We note that the magnetic grain size distribution from HRTEM images shows an average particle diameter of 10 nm (see Supplementary Fig. S11). This is congruous with TEM analysis from a study of Shanghai subway PM. They observed ‘clumped’ submicron-sized Fe-rich particles, which was consistent with the presence of SP and SD grains revealed by magnetic techniques^33^. Fe-rich particles observed in this study are similar to TEM images of outdoor vehicular brake wear PM, where a prolific number of 10–50 nm sized nanoparticles form larger agglomerates^34^, conversely to a study in Rome^35^ where it was concluded that SP particles (ultrafine particles < 30 nm) occur as a result of stress in the oxidized outer shell of MD particles (particles > 700 nm). Moreover, in vehicle braking systems, magnetic PM emissions are dominated by magnetite^34,36–38^ ,however, our results suggests that the rail-wheel-brake wear airborne particles in the LU are dominated by maghemite (see Supplementary Fig. S4).

The PM particle-size distribution observed here can be explained by the wear mechanism of the wheel-rail contact, which itself is a function of normal load or sliding velocity^39^. A railway PM study established that higher load on trains increased PM generation within 0.25–1 μm particle size interval^39^. Another train PM study observed metallic particles as fine as 50 nm in diameter generated by train disc brake abrasion. They also found that higher speeds and corresponding higher braking temperatures produced finer sized particles (280 nm) than the usually dominant 350 nm diameter sized particles at 70 km/h^40^.

Our results complement existing data on magnetic PM of underground rail and confirm the abundance of ultrafine Fe-bearing particles. Traditional PM monitoring classifications in terms of PM_2.5_ or PM_10_ etc. may underestimate the presence of ultrafine particles. We note that although we find particles up to 500 nm in diameter, the majority of particles are very fine, and only appear larger when they are agglomerated. The similarity in FORC signals (no MD signal, relatively little V/PSD signal) and coercivity distributions demonstrate that the bulk magnetism is not drastically different when we compare PM_10_, PM_4_, and PM_2.5_ filters. In fact, primarily the particles are < 0.1 μm—thought to be the most dangerous as they have a higher propensity to be translocated into the bloodstream from lungs than larger particles. Our FORC and low-temperature measurements are consistent with range of particle sizes backed up by high-resolution microscopy and tomography, providing confidence that magnetic characterisation techniques give an accurate and rapid assessment of the true nature of the Fe-oxide particles present. Previous subway studies have found either metallic Fe^26,33^, magnetite^26,33,41^, maghemite^26,42^ or hematite^42^ to be the main Fe-oxide phase. A study comparing the genotoxicity of Stockholm subway particles found that subway PM (which mainly consisted of magnetite) was more genotoxic than other particle types. By comparing synthetic magnetite particles with subway PM, they found subway PM caused mitochondrial depolarization and DNA damage which was not explainable by similar experiments done on magnetite particles. Since genotoxicity could not be explained by the most abundant component—magnetite; they concluded that the genotoxicity is most likely governed by highly reactive surfaces that cause oxidative stress^41^.

Apart from the different toxicity of magnetite and maghemite, we believe the oxidation state of Fe-oxide particles can be also potentially used as a measurement of PM maturity where one could possibly differentiate the proportion of freshly generated PM (less exposed to air) to older resuspended PM (more exposed to air). Our study suggests that particle size classification is important where, for example, PM_2,5_ concentrations include large clusters of particles that are 0.01 μm in diameter. This could have implications on what percentage of this ‘PM_2.5_’ can be deagglomerated in lungs and make its way into our blood stream. The indoor setting of the LU means that outdoor air pollution regulations do not apply. However, our results suggest that high PM levels in such microenvironments should be as strictly regulated as any other busy urban street in London. Since LU PM originates primarily from tube operations, the amount of resuspended dust in the system could be reduced by removing accumulated dust on the tracks, washing the tracks and walls of the tunnel^43^, using magnetic filters in ventilation systems to remove magnetic PM^44^ or placing screen doors between the platform and train to reduces PM exposure on platforms^45^.

## Conclusions

A combination of magnetic and microscopy techniques unravels nature of Fe-oxide nanoparticles in the London Underground. We found that agglomerates of Fe-oxide nanoparticles comprised of individual 5–20 nm particles or discrete particles between 20 and 500 nm in diameter. Our methods compliment traditional monitoring systems which in this microenvironment would (1) underestimate the number of ultrafine particles (2) skew the particle size distribution to large particles (as particles are aggregated). Most of these particles are ‘aged’ and resuspended maghemite, which means they have been in the microenvironment ambient air for longer times. The size, morphology, and composition of these particles can help us constrain the reasons which govern health implications of Fe-oxide particles.

## Materials and methods

### Air filter samples

Transport for London (TfL) provided us with respirable dust samples from platforms, ticket halls, and train operator cabins collected using air quality monitoring instruments in two different monitoring campaigns.

In the first campaign, only respirable dust (RD) (PM_4,_ < 4 μm) samples were collected at different platforms, and ticket halls (see Supplementary Table S2). Personal samplers equipped with 25 mm GLA 5000 polyvinyl chloride (PVC) filters were given to TfL staff whilst on duty. Alternatively, where the staff were not comfortable with carrying personal monitors, static sampling pumps were set up at the headwalls (where the first train arrives). For the second train operators’ exposure monitoring campaign, sized fractionated samples were collected at a flow rate of 2 l/min within the cabs of nine LU train lines using a static SKC Personal Environment Monitor equipped with PM_2.5_ and PM_10_ impactor heads (see Supplementary Tables S2 and S3). Further details on sampling method provided in Supplementary Text S1.

### Magnetic characterisation

Magnetic remanence measurements were done at the Centre for Environmental Magnetism and Paleomagnetism (CEMP), Lancaster University. A Molspin demagnetiser was used to impart Anhysteric Remanent Magnetisation (ARM) by applying 80 mT alternating field (AF) and 100 μT direct current (DC) bias field (ARM_80/100,_ also known as χ_ARM_). The samples were subsequently alternating-field (AF) demagnetized at 5, 10, 15, 20, 25, and either 30 mT, 40 mT, or 60 mT fields. The AF demag field that caused the ARM value to halve defines the median destructive field (MDF_ARM_). Room-temperature isothermal remanent magnetization (IRM) was acquired at 20 and 100 mT using a Molspin pulse magnetizer, 300 mT and 1000 mT using a Newport electromagnet.

### Hysteresis, first order reversal curves (FORCs), ZFC–FC curves, temperature dependent susceptibility

Room-temperature hysteresis loops, direct-current demagnetisation (dcd) curves, FORCs^46,47^ and remFORCs^23^ were measured using a Princeton Micromag Alternating Gradient Magnetometer (AGM) at the Department of Earth Sciences, University of Cambridge. A total of 513 FORCs were acquired in discrete mode for each sample at a field step of 1 mT and an averaging time of 300 ms. FORC diagrams were processed with the FORCinel software^48^ using VARIFORC smoothing^49^. Backfield remanent coercivity distribution (− dM/dlog(B_c_)), also defined as the first derivative of direct current demagnetisation (dcd) curve, was obtained directly from the corresponding FORC diagrams (Fig. 1A). Low-temperature (LT) magnetic behaviour of the particles was analysed on a Quantum Design (QD) Magnetic Property Measurement System (MPMS3) at the Maxwell Centre, University of Cambridge. In addition, a total of 80 FORCs were measured at 10 K and 300 K on sample 202073-511 using the ‘xFORC for QD’ sequence generator from^22,23^. To better understand grain size, and identify ferromagnetic minerals based on low-temperature transitions^27^, we measured zero-field cooling and field cooling (ZFC–FC) curves and room-temperature saturation isothermal remanent magnetisation (SIRM) warming and cooling curves on the MPMS3 using the sequence based on^18^; protocol details are provided in the Supplementary Text S1. A quantitative estimate of superparamagnetic (SP) grain fraction in air filter dust is calculated as the ratio (LT-SIRM_10K(ZFC)_ – RT-SIRM_10K_)/LT-SIRM_10K(ZFC)._

Frequency (λ_FD_ %), and temperature-dependent susceptibility measurements from 1 Hz through to 150 Hz for temperatures between 300 and 100 K were measured on a Lakeshore Vibrating Sample Magnetometer (VSM) at the Institute of Rock Magnetism (IRM), University of Minnesota.

High temperature-dependent susceptibility measurement was performed using an AGICO Kappa bridge with a CS-4 high-temperature furnace at Department of Earth Sciences, University of Cambridge. Susceptibility was measured as the air filter sample was heated from 40 to 700 °C and then cooled back to 40 °C at an operating frequency of 976 Hz. The experiment was carried out in an argon environment to prevent any oxidation.

### Electron microscopy analysis

Samples for TEM were prepared by placing the PVC air filters in a 5 ml Eppendorf tube and ultrasonicating the particles for 30 s in distilled water. A disposable pipette was used to take a few drops of the suspended solution onto a copper TEM grid and was left to dry. SEM was performed directly on the TEM grids sitting on an Al stub (see Supplementary Fig. S9) at the University of Cambridge using a Thermofisher Quanta-650F SEM equipped with a backscatter electron (BSE), a secondary electron (SE), and an energy-dispersive spectroscopy (EDS) detector. Segmentation of the chemical maps and unmixing of overlapping chemical spectra was performed using a machine-learning autoencoder technique^50^. This procedure was used to provide a low-resolution overview of the PM and to identify representative regions of interest for further investigation using an FEI Tecnai F20 FEG TEM. High-resolution TEM (HRTEM) images, selected area electron diffraction patterns (SAED) and EDS maps in scanning transmission electron microscopy (STEM) mode were acquired.

Electron tomography was performed using an FEI Krios TEM operating at 300 keV and a temperature of 80 K at the University of Cambridge. The sample was prepared the same way as our TEM analysis and we acquired a total of 100 high-angle annular dark-field (HAADF) images as a function of sample tilt angle, with steps of 1.5° between ± 60° and steps of 1° from ± 60° to ± 70°. Tomographic reconstruction was performed using a compressed sensing algorithm^51^. Further details are provided in the Supplementary Text S1.

## Supplementary Information

Supplementary Information.

## Data Availability

All data generated or analysed during this study are included in this published article (and its Supplementary Information files).
