# Supplementary material for: Magnetic and microscopic investigation of airborne iron oxide nanoparticles in the London Underground
Source: Sci Rep. 2022 Dec 15;12:20298. doi: 10.1038/s41598-022-24679-4 (PMC9755232; doi:10.1038/s41598-022-24679-4)
Supplement: Supplementary file 1 — Supplementary Information. [file 41598_2022_24679_MOESM1_ESM.pdf]

Supplementary Information for

London Underground air pollution particles are finer than you think

H. A. Sheikh[1*], P.Y. Tung[1,2], E. Ringe[1,2], R.J. Harrison[1]

[1]Department of Earth Sciences, University of Cambridge, Cambridge, UK

[2]Department of Materials Sciences, University of Cambridge, Cambridge, UK

*has57@cam.ac.uk

This file includes:

Supplementary text S1

Figures S1 to S12

Tables S1 to S3

SI References

Supplementary Information Text

Methods

Sampling campaign. The first sampling campaign was conducted between 15th February 2019 to 4th March 2019. The samples we looked at included personal monitors on station staff conducting gate line duties or static monitors at platforms (see Supplementary Table S2 for locations of the samples analyzed). The air was sampled for 4 hours, and the dust concentration was normalized to an 8-hr time-weighted average (TWA) concentration for respirable dust. The dust concentrations reported in Supplementary Table S2 are TWA concentrations equivalent to 8-hr exposure in an 8-hr period, for example, 4-hr exposure in an 8-hr period, the TWA is 1/2 of the level measured for the 4-hr period. The second sampling campaign was undertaken over three working shifts between 21st April and 8th July 2021 on each of the nine LU tube lines using SKC Personal Environmental Monitor $PM_{2.5}$ and PM_{10} impactor heads with glass fibre filters**.** The train operator duty times determined how long the air is sampled which varied between 3-6 hours, and the concentrations are reported in TWA. Route and duty details for the trains are presented in Supplementary Table S3.

LT-SIRM data acquisition. Each sample was wrapped in a cling film and inserted into a gel cap before measuring on the magnetic property measurement system (MPMS). The protocol was sequence was based on[1] and is as follows (a) a room temperature SIRM (RT-SIRM) was imparted in a 2.5 T field and then measured on cooling from 300 to 10 K; (b) the RT-SIRM was then measured on warming back to 300 K; (c) the sample was zero-field cooled (ZFC) to 10 K and a low-temperature SIRM (LT-SIRM) imparted in a 2.5 T field; (d) the ZFC LT-SIRM was measured on warming to 300 K; (e) the sample was then field cooled (FC) in 2.5 T from 300 to 10 K and the resulting FC LT-SIRM was measured from 10 to 300 K.

Tomographic reconstruction. We manually removed frames that were obscured at high angle. The first 13 frames of region 5 (Fig. 4A), last 15 frames of region 11 (Fig. 4B), and last 4 frames of region 12 (Fig. 4C) were not usable, therefore, were removed. We then performed image-shift alignment on Sobel-filtered images using a phase correlation algorithm and tilt-axis alignment using a manual procedure to minimise arcing in the reconstructions**.** We ran a total of 1000 iterations for all three reconstructions of Chambolle-Pock algorithm[2] with a weighting of 0.05 for the total variation regularisation term. Dragonfly software was then used to perform intensity thresholding and segmentation of particles (see Fig. 4).

LT-SIRM Sequence
New Datafile "C:\QdSquidVsm\Data\Hassan has57\86\1.dat"
Set Magnetic Field 25000.0Oe at 500.00Oe/sec, Linear, Stable
Wait For Temperature, Field, Delay 300 secs (5.0 mins), No Action
Set Magnetic Field 0.0Oe at 500.00Oe/sec, No O'Shoot, Stable
Wait For Temperature, Field, Delay 300 secs (5.0 mins), No Action
Magnet Reset
Wait For Temperature, Field, Delay 1200 secs (20.0 mins), No Action
DC Measure 10 scans over 35 mm in 4 sec Auto-Tracking
Wait For Temperature, Field, Delay 60 secs (1.0 mins), No Action

New Datafile ""C:\QdSquidVsm\Data\Hassan has57\86\2_RT-SIRM_cooldown_in_ZF.dat"
MPMS3 Moment (DC) vs Temperature 300K to 10K Sweep Continuous Auto-Tracking
Wait For Temperature, Field, Delay 60 secs (1.0 mins), No Action

New Datafile ""C:\QdSquidVsm\Data\Hassan has57\86\3_RT-SIRM_warm_up_in_ZF.dat"
MPMS3 Moment (DC) vs Temperature 10K to 300K Sweep Continuous Auto-Tracking
Wait For Temperature, Field, Delay 60 secs (1.0 mins), No Action

Set Temperature 10K at 35K/min. Fast Settle
Wait For Temperature, Field, Delay 600 secs (10.0 mins), No Action

New Datafile " C:\QdSquidVsm\Data\Hassan has57\86\4_10K_remanence.dat"
Set Magnetic Field 25000.0Oe at 500.00Oe/sec, Linear, Stable
Wait For Temperature, Field, Delay 300 secs (5.0 mins), No Action
Set Magnetic Field 0.0Oe at 500.00Oe/sec, No O'Shoot, Stable
Wait For Temperature, Field, Delay 300 secs (5.0 mins), No Action
Magnet Reset
Wait For Temperature, Field, Delay 1200 secs (20.0 mins), No Action
DC Measure 10 scans over 35 mm in 4 sec Auto-Tracking
Wait For Temperature, Field, Delay 60 secs (1.0 mins), No Action

New Datafile " C:\QdSquidVsm\Data\Hassan has57\86\5_LT-SIRM_warm_up_in_ZF.dat"
MPMS3 Moment (DC) vs Temperature 10K to 300K Sweep Continuous Auto-Tracking
Wait For Temperature, Field, Delay 60 secs (1.0 mins), No Action

Set Magnetic Field 25000.0Oe at 500.00Oe/sec, No O'Shoot, Stable
Wait For Temperature, Field, Delay 600 secs (10.0 mins), No Action

New Datafile " C:\QdSquidVsm\Data\Hassan has57\86\6_MvT_cooldown_in_25000Oe.dat"
MPMS3 Moment (DC) vs Temperature 300K to 10K Sweep Continuous Auto-Tracking
Wait For Temperature, Field, Delay 60 secs (1.0 mins), No Action

New Datafile " C:\QdSquidVsm\Data\Hassan has57\86\7_10K_remanence_after_FC.dat"
Set Magnetic Field 0.0Oe at 500.00Oe/sec, No O'Shoot, Stable
Wait For Temperature, Field, Delay 300 secs (5.0 mins), No Action
Magnet Reset
Wait For Temperature, Field, Delay 1200 secs (20.0 mins), No Action
DC Measure 10 scans over 35 mm in 4 sec Auto-Tracking
Wait For Temperature, Field, Delay 60 secs (1.0 mins), No Action

New Datafile " C:\QdSquidVsm\Data\Hassan has57\86\8_LT-SIRM_warm_up_in_ZF_after_FC.dat"
MPMS3 Moment (DC) vs Temperature 10K to 300K Sweep Continuous Auto-Tracking
Wait For Temperature, Field, Delay 60 secs (1.0 mins), No Action

Results (technical description)

Temperature dependent magnetic measurements
We compare low-temperature (LT-SIRM$_{10K}$) with room temperature (RT-SIRM$_{300K}$) to quantify the contribution from SP particles that have blocking temperatures below room temperature (see Methods and materials for the equation). RT-SIRM$_{300K}$ for sample 511 (Operator cabin, Northern line, PM$_{2.5}$) represents 23% of the LT-SIRM$_{10K}$, meaning the remaining 77% of LT-SIRM is carried by SP particles at 300 K that become progressively blocked as the sample is cooled to 10 K (see Supplementary Fig. S2). For platform samples 180487-56 (Platform 7 Jubilee line, Baker Street Station), 180487-58 and 180487-96 (Platform 4 Bakerloo line, Paddington Station) 60-64% of LT-SIRM$_{10K}$ is carried by SP particles (see Supplementary Fig. S2). Field-cooled and zero-field cooled (FC-ZFC) remanence warming curves and RT-SIRM warming, and cooling curves (see Supplementary Fig. S2) do not show any evidence of a Verwey transition or dampened transition, which is usually observed by a loss in remanence at temperatures 80-125 K upon warming.

The frequency-dependent susceptibility (λ_{FD} %) varies between 0 to 6.5% and can be used as an indicator of particles near the SP/SD threshold (see Supplementary Fig. S3). The in-phase (real) component of susceptibility χ′ reduces with an increase in frequency while out-of-phase component of magnetic susceptibility (imaginary), χ″, shows a peak shift to lower temperatures with decreasing frequency (see Supplementary Fig. S3).

High temperature-dependent susceptibility measurement for sample 180487-87 (Oxford Circus, Central line W/B, PM$_4$) showed an irreversible decrease in susceptibility between 206ºC and 460ºC is characteristic of maghemite – the fully oxidised, metastable form of magnetite that transforms to

hematite irreversibly on heating above 200°C (see Supplementary Fig. S4), similar to diagnostic curves in a previous study[3]. There is no evidence of drop in susceptibility at 565ºC associated with magnetite on heating; a small increase below 565ºC on cooling is observed, suggesting the formation of small amount of magnetite at high temperatures (argon creates reducing conditions).

Room temperature magnetic granulometric measurements

Room-temperature χ_{ARM}/SIRM ratio versus MDF_{AF} is plotted in Supplementary Fig. S1. χ_{ARM}/SIRM ratio has been used as an indicator for grain size variation for samples with homogeneous mineralogy[4,5]. The bulk magnetic properties lie close to the line defined by uniform-sized, non-interacting magnetite in the size range 1-7.5 μm (see Supplementary Fig. S1).

First order reversal curves (FORCs)

FORCs were measured for different localities within the LU (Fig. 1 B, C, and D) and for different PM size fractions (PM_{10}, PM_4, and PM_1) and corresponding coercivity distributions (Fig. 1A) were calculated. The peak of the derivative of the backfield remanence curve (i.e., the coercivity distribution) occurs at an identical position (B_c = 65 mT) for all samples, and the distribution decays to zero by ~300 mT. The shape of the coercivity distribution is very similar in all air filter samples, with variations in the height of the peak caused by variations in the proportion of SP particles. We performed FORC Principal Component Analysis (FORC-PCA)[6] on our processed FORCs to test any 'fingerprint' variation between samples (Fig. 1G). All our PM samples lie between two identified endmembers (EM), which contain broadly similar features expressed to subtlety varying degrees. The magnetic signature of EM1, which primarily, but not exclusively, encompasses PM_4 filter samples from the platform and ticket halls, exhibits: (1) an SD central ridge (particles between 30 nm and 70nm) at B_u=0 extending to >200 mT; (2) a clear vortex/pseudo-single domain (V/PSD) component (particles diameter between 70-700 nm); and (3) a vertically asymmetric signal at the origin that is consistent with the presence of superparamagnetic (SP) particles (nanoparticles <30 nm in diameter). Similar features are observed in EM2 (consisting primarily, but not exclusively, of $PM_{2.5}$ and PM_{10} air filters from train operator cabins) but with relative greater intensity for the SP component and weaker intensity for the SD and V/PSD and signals compared to EM1.

To observe changes in FORC fingerprint at low temperatures, LT-FORCs were measured at 10 K on sample 180487-511 (Northern line, $PM_{2.5}$ filter). At room temperature, the FORC diagram shows the presence of an SP signal and SD ridge extending to 200 mT. At 10 K, the coercivity shifts to a higher coercivity of 250 mT along the B_c axis and broadens significantly in the B_u axis. An SP signal at B_c = 0 is still observable, indicating the presence of SP particles (<<30 nm in diameter) with blocking temperatures below 10 K.

Remanence FORCs (remFORCs) were measured using the irregular measurement algorithm devised by[7,8] for sample 180487-58 (Baker Street, Bakerloo line, PM_4 filter). The conventional FORC showed the same mix of SP, SD and V/PSD as the regular FORC measurements; the remFORC highlights both the SP and SD contributions. Here, the SP signal is isolated in the remFORC diagram near the SP to SD threshold size as that region is sensitive to viscous magnetization processes[8] , the transient FORC (tFORC) (see Supplementary Fig. S5) shows the transient hysteresis responses[9] related to vortex nucleation and annihilation processes, and highlights the lack of a clear MD signal (particles > 700 nm); the induced FORC (iFORC) diagram illustrates a noisy, but just visible, negative-positive-negative-positive (NPNP) signal that is related to vortex state or strongly interacting particles[10] (see Supplementary Fig. S5).

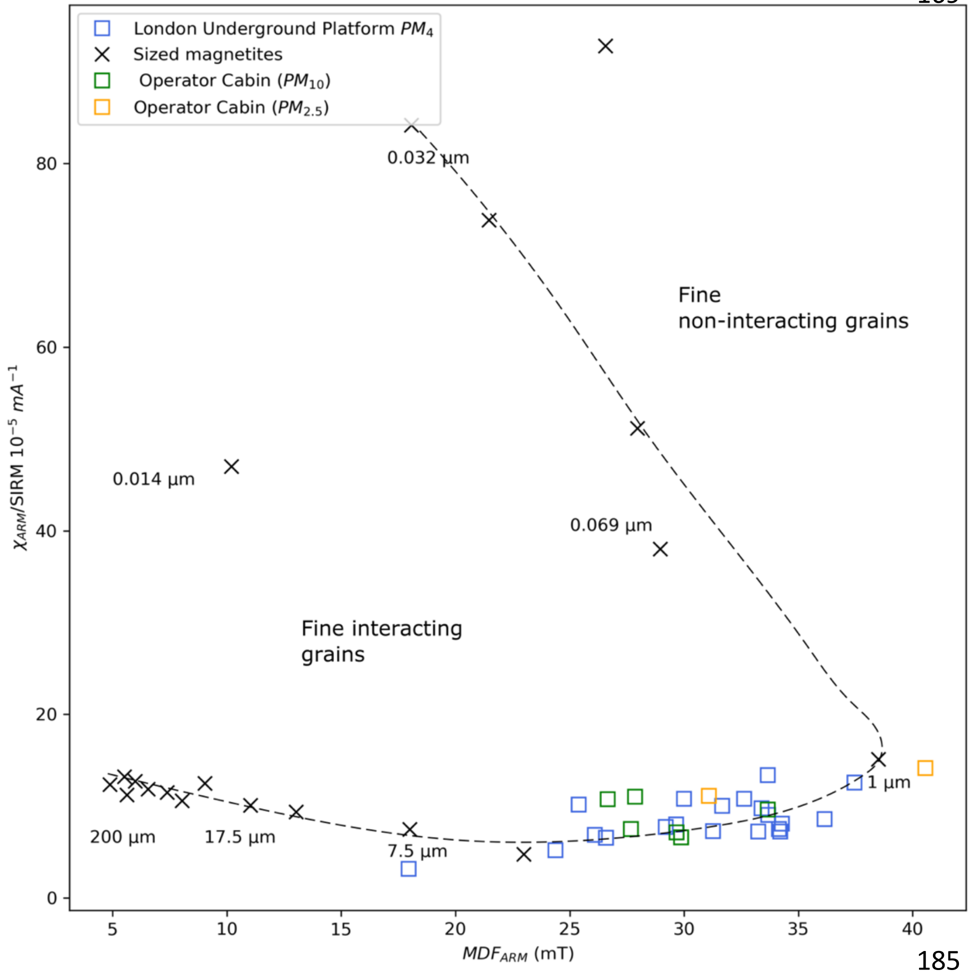

Figure S1. Bulk room temperature magnetic granulometric measurements presented on a classic sized-magnetite measurements and is indicative of mean grain size[4,5]. On y-axis we have plotted a ratio of room temperature anhysteretic remanent magnetization (ARM) susceptibility (χ_{ARM}) normalized by saturation isothermal remanent magnetization (SIRM). On x-axis, the ARM mean destructive field (MDF_{ARM}) of each sample is plotted, which is defined when the magnetic fraction loses half of its remanent magnetization.

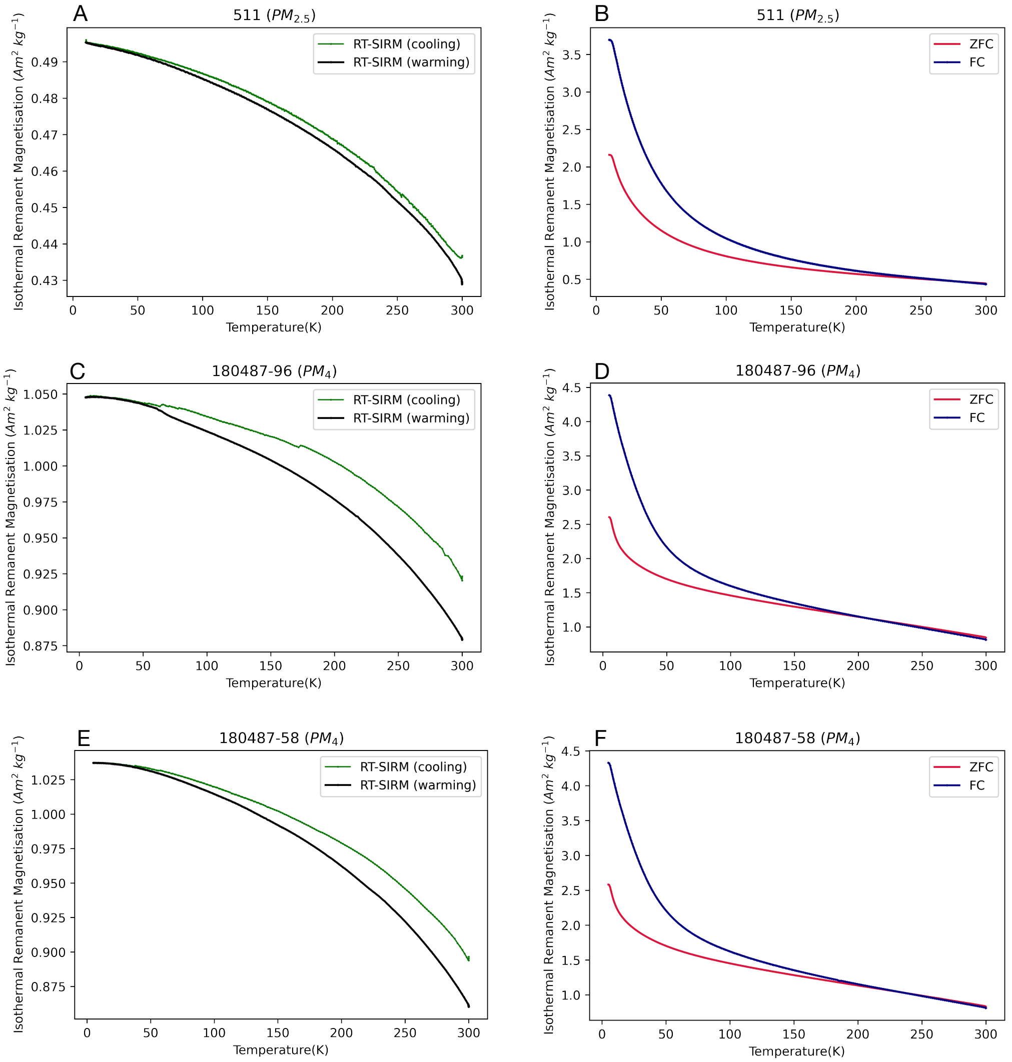

Figure S2. Low-temperature remanent magnetization curves for four different samples from different localities within the London Underground. Room temperature saturation isothermal remanence (RT-SIRM) warming, and cooling curves (A, C, E) do not show any evidence of Verwey transition at 120-125 K or lower K, hinting the sample has had time to oxidize sufficiently to suppress the transition. Zero-field cooled, and field cooled (ZFC and FC) curves (B, D, F) also do not show any evidence for Verwey transition or dampened transition (usually seen for surface oxidized magnetite).

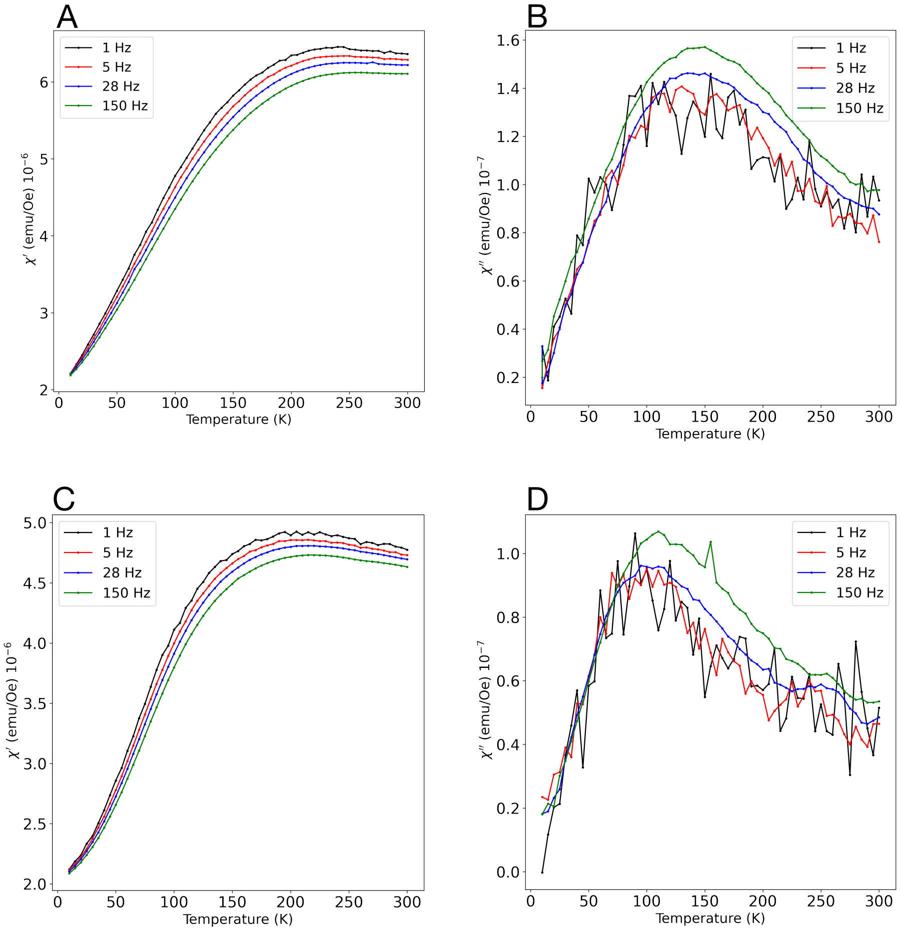

Figure S3. Frequency dependent curves as a function of temperature. **(**A and C): At each temperature, in phase (real) susceptibility curves at frequencies f= 1.0, 5.3, 28.3, 150.5 to 800.5 Hz for sample 180487-56 and 180487-92 respectively, (B and D): Out of phase susceptibility (imaginary) curves at each temperature. (E and F) frequency dependent susceptibility calculated by subtracting susceptibility at 150 Hz from 1.0 Hz.

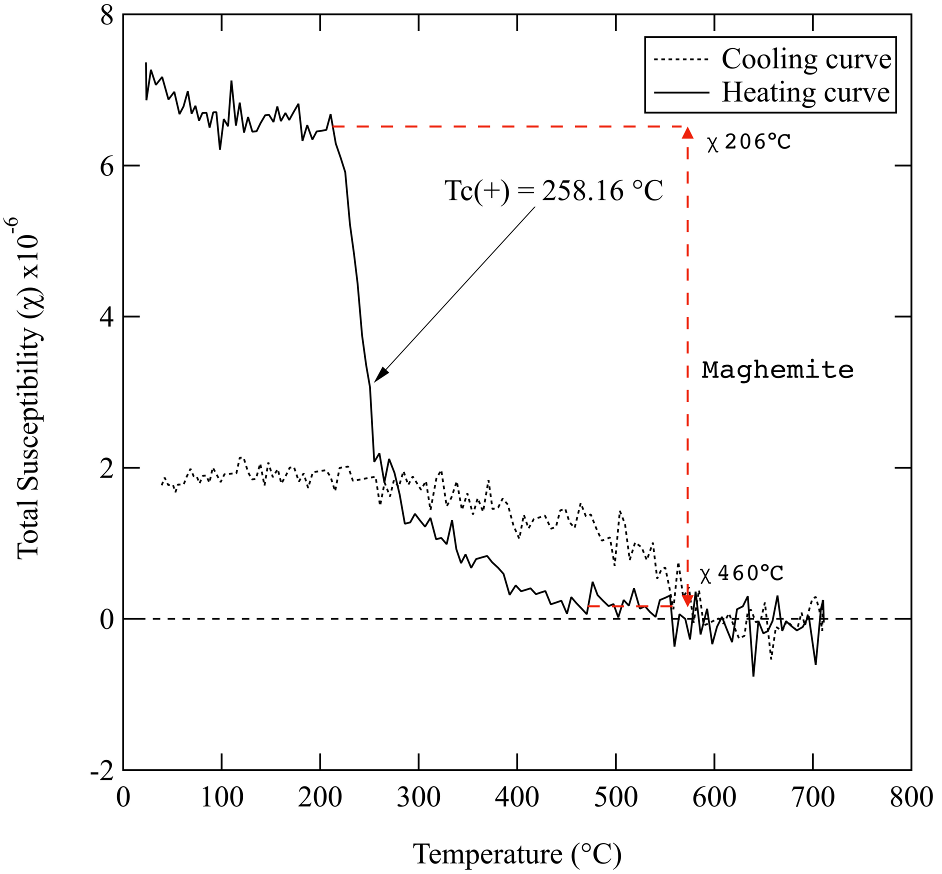

Figure S4. High temperature-dependent susceptibility for sample 180487-87. During heating, there is an observed susceptibility difference between temperature at 206ºC and 460ºC—diagnostic of maghemite. In cooling, a slight increase in susceptibility is observed at around 580ºC, which is probably a result of hematite being reduced to magnetite and is not a primary mineral present.

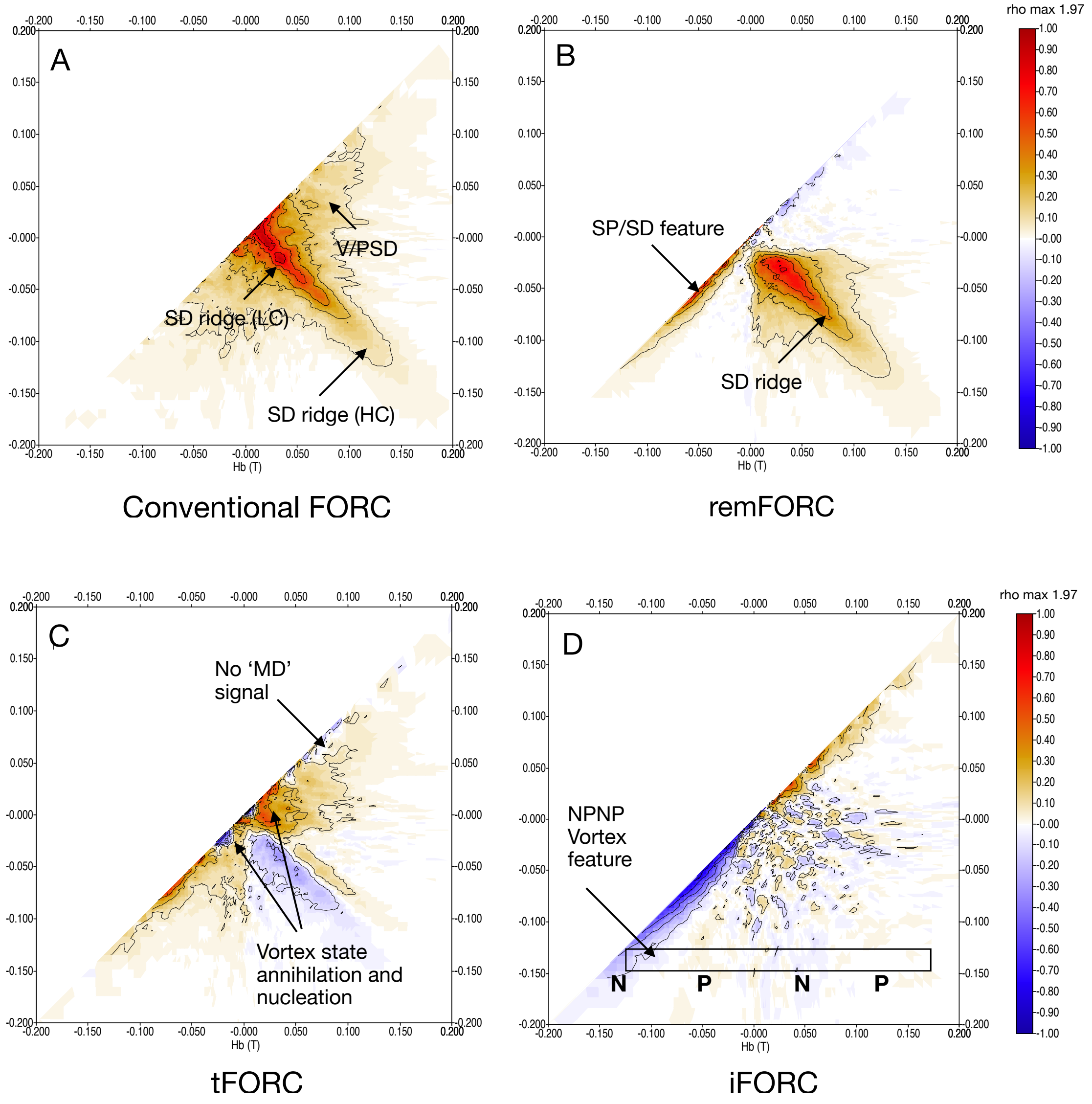

Figure S5. Domain state signals from conventional FORC and FORC-type diagrams of [8] from sample 180487-58. (A) a conventional FORC diagram shows a SD and a Vortex/PSD signal. (B) signal from remanence-bearing particles includes an interacting SD component, and a component near the SP/SD threshold that is visible in the negative B_u axis. (C) The transient (tFORC) diagram shows absence of an 'MD' wing and upper and lower lobes of the diagram represent vortex state annihilation and nucleation. (D) The induced (iFORC) diagram shows weak evidence of the negative-positive-negative-positive (NPNP) signal associated with vortex and/or interacting SD particles.

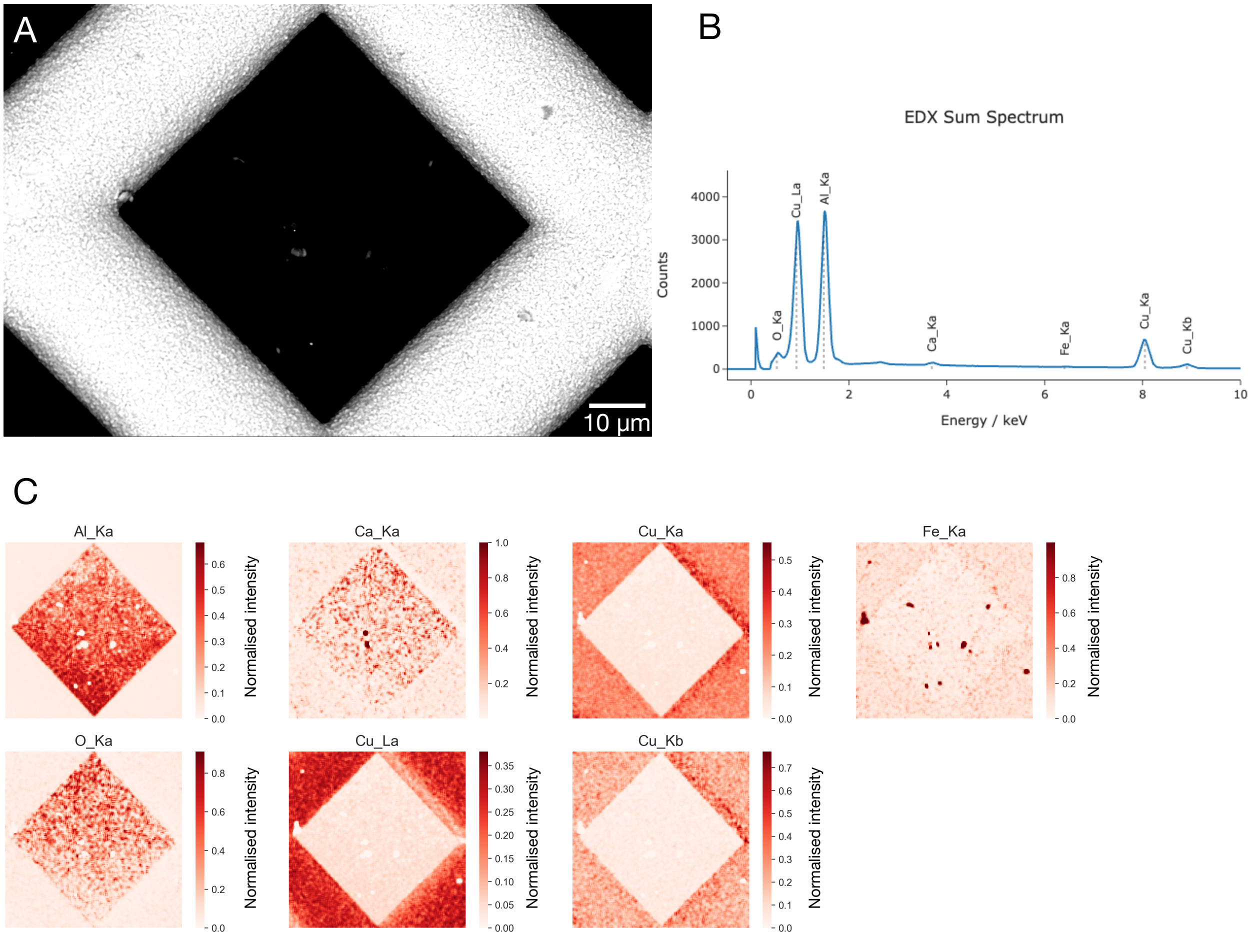

Figure S6. Scanning electron microscopy (SEM) (A): Zoomed out BSE image of the TEM grid showing sparse clusters of Fe-oxide. (B): EDS sum spectra of the region showing strong signals of Al and Cu which are coming from the stub and the grid respectively. (C) confirms the Al signal is from scattering of electrons from the TEM stub holder for the SEM, and also confirms the presence of Fe-rich clusters.

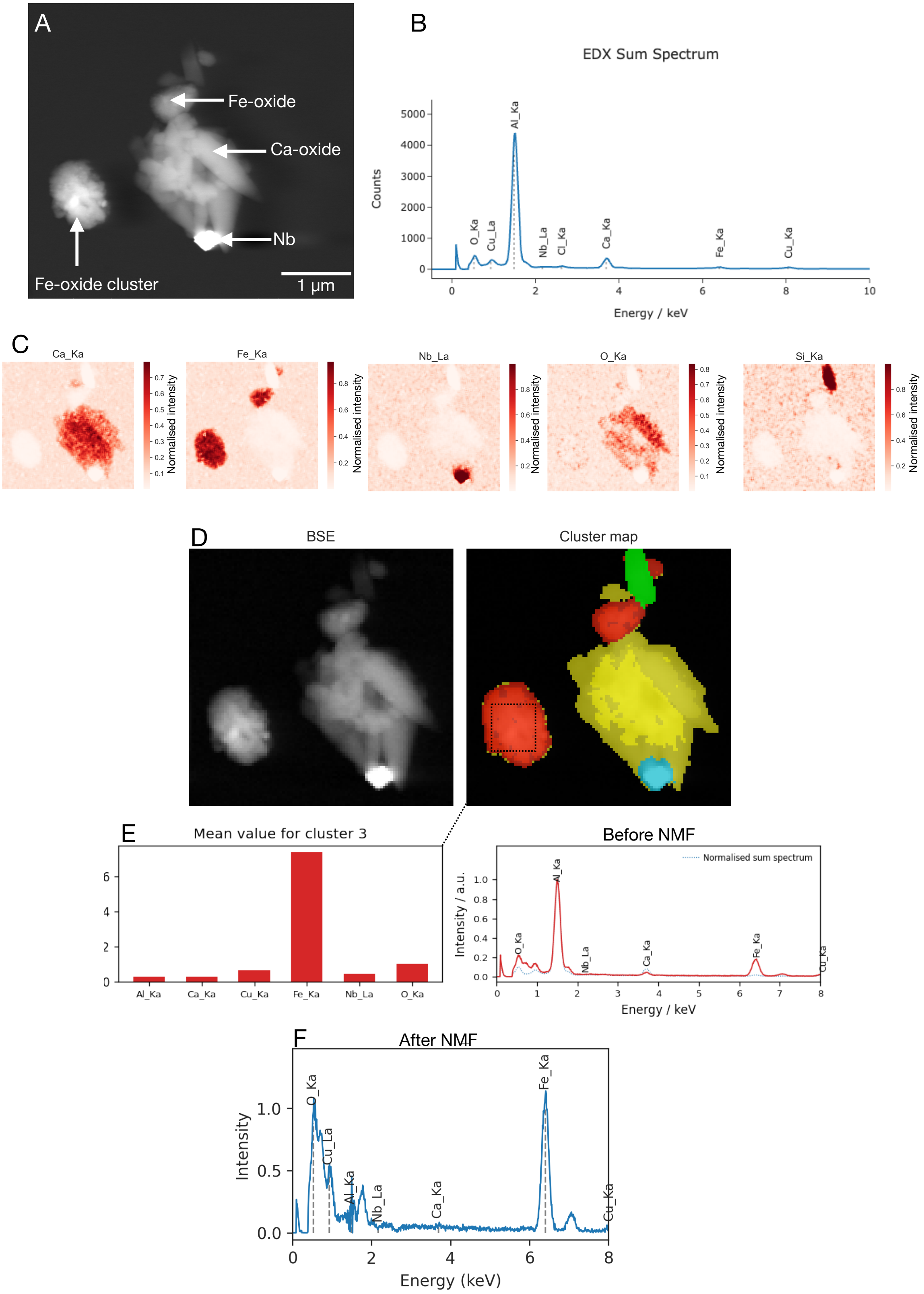

Figure S7. Scanning electron microscopy (SEM). Detailed textural and chemical analysis using backscatter electron (BSE) image and elemental maps produced using energy dispersive x-ray spectroscopy (EDS). (A) shows the associated BSE image; (B) EDS spectrum; (C) elemental maps for the region of interest, (D) BSE image and respective cluster map showing at least 4 identifiable phase; Red: Fe-rich, Yellow: Ca-rich, Green: Si-rich, and Blue: Nb-rich (E) Soft-max normalised signal[11] for

each element where strong signal shows that elements are present at above-average levels and EDS spectrum of cluster 3 (Fe-rich phase); (F) EDS spectrum after performing non-negative matrix factorisation (NMF) on the identified clusters removes the dominant background peak due to the Al SEM stub.

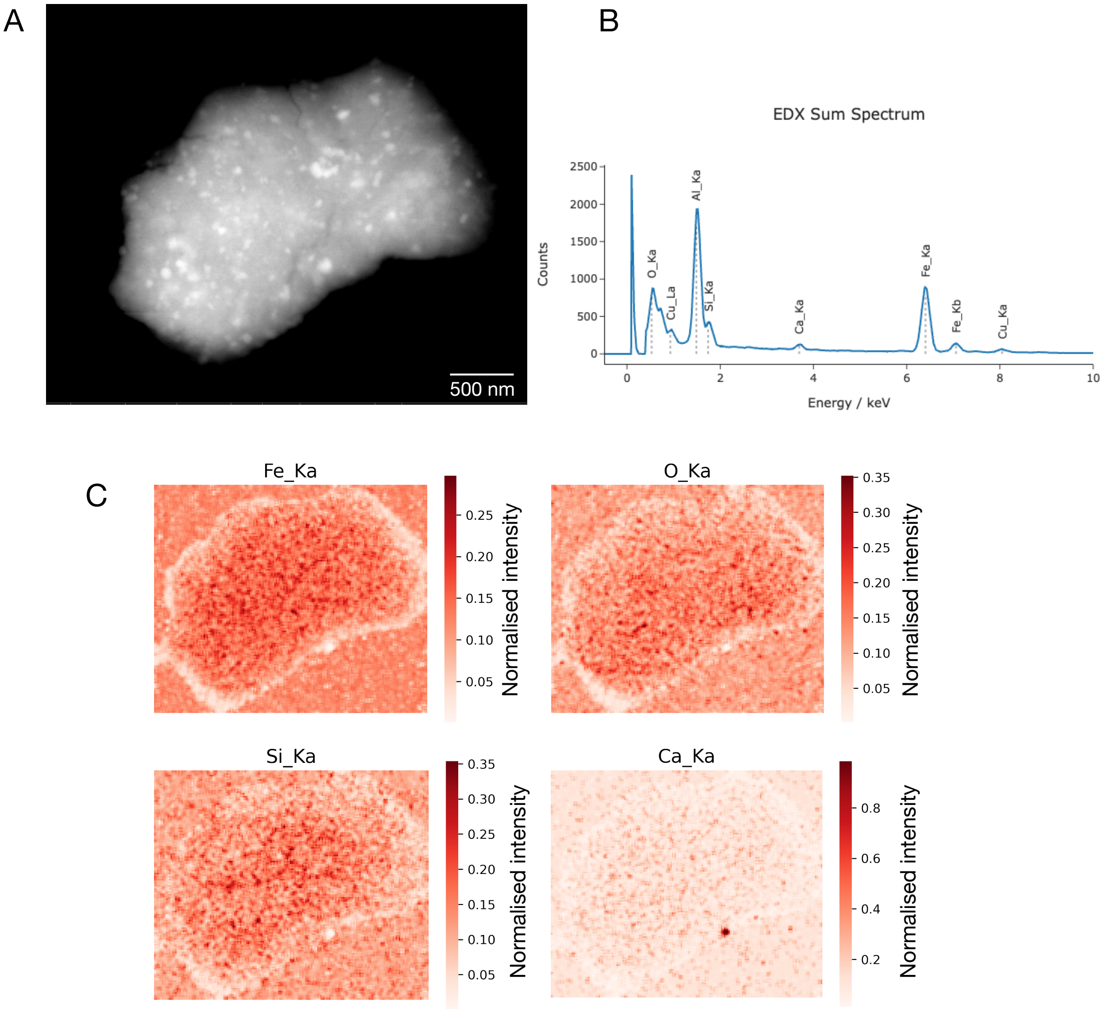

Figure S8. Scanning electron microscopy (SEM) (A) BSE image of an Fe-oxide particle cluster, (B) sum spectrum of elemental count from region A, (C) elemental maps showing presence of Ca-rich signal from a particle in the background.

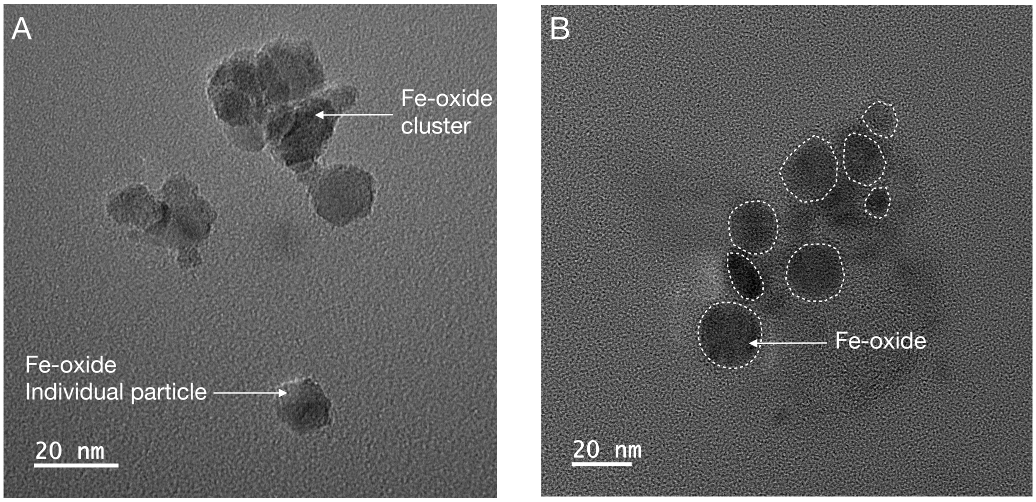

Figure S9. Transmission electron microscopy (TEM) images of sample 180487-86 showing (A) isolated Fe-oxide nanoparticles and clusters of Fe-oxide nanoparticles (B) showing interacting individual nanoparticles.

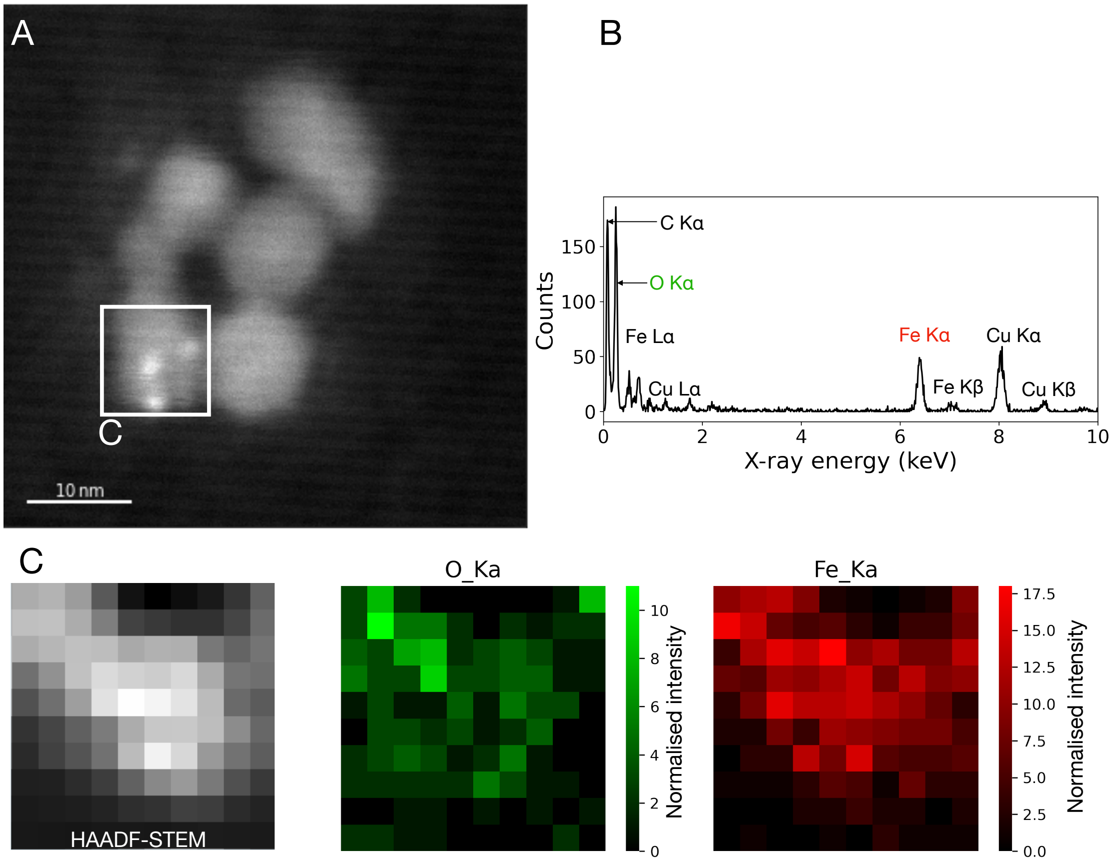

Figure S10. Morphological and chemical fingerprints of representative Fe-bearing particles from London Underground. (A) HAADF-STEM imaging of spherical Fe-O nanoparticles. (B) EDS spectrum of the marked region. (C) HAADF and EDS chemical maps of the marked region, confirming strong signals from Fe and O, consistent with particles examined using the SEM-EDS.

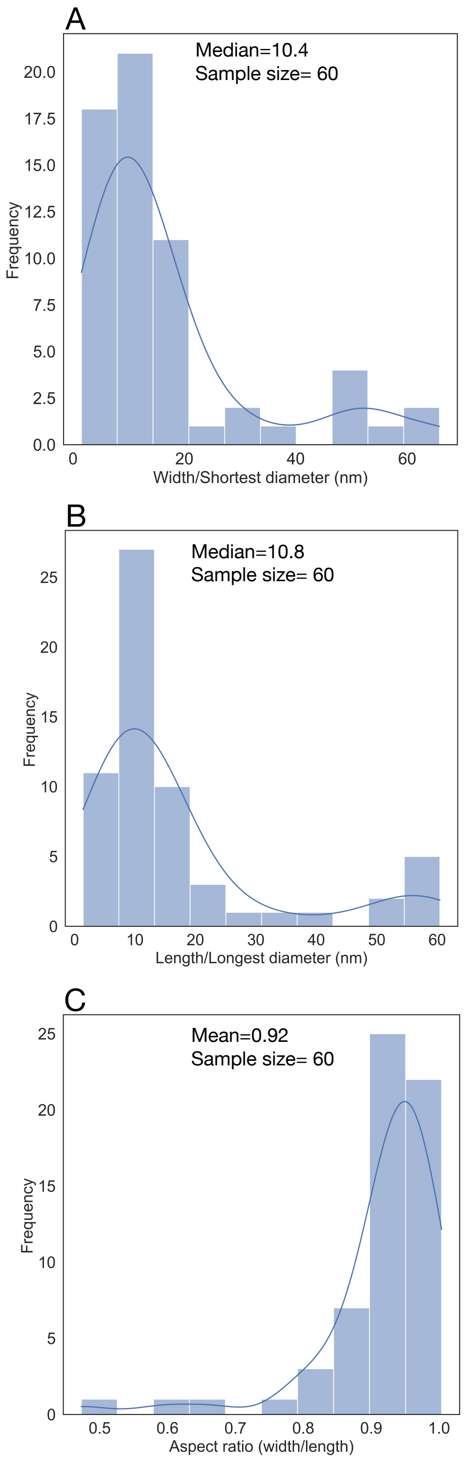

Figure S11. Quantification of particle sizes done by individually measuring particle diameters from clusters in HRTEM images. (A) shortest diameter (B) longest diameter (C) aspect ratio i.e., the circularity of particles measured as a ratio of width/length (a value of 1.0 is a perfect sphere).

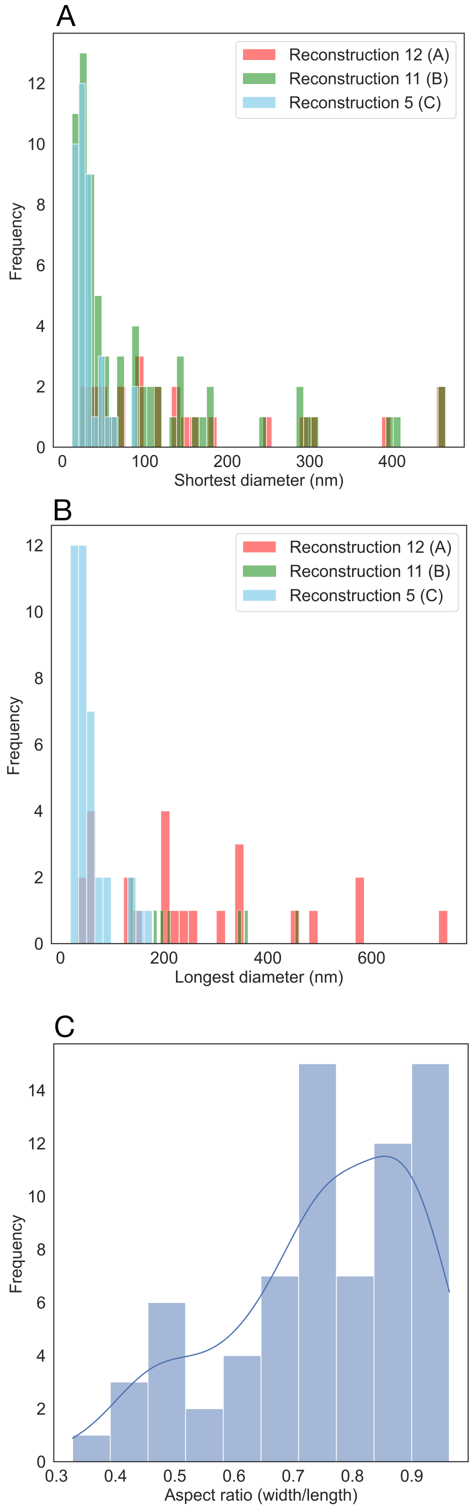

Figure S12. Quantification of particle sizes from segmented 3D tomography data on dragonfly. (A) shortest diameter of particle (B) longest diameter of particle (C) aspect ratio i.e., the circularity of particles measured as a ratio of width/length (a value of 1.0 is a perfect sphere. Green: Reconstruction-region-12; Red: Reconstruction-region-11; Blue: Reconstruction-region-5.

Table S1. Dimensional measurements for Fe-oxide particles observed under transmission electron microscopy (TEM) of PM-loaded air filters.

TEM sample	Mode	Image	Length	Width	Shape factor (width/length)
180487-86	TEM	1	13.42	7.93	0.59
180487-86	TEM	1	12.77	11.6	0.91
180487-86	TEM	1	9.54	8.16	0.86
180487-86	TEM	1	3.69	3.46	0.94
180487-86	TEM	1	7.62	7.02	0.92
180487-86	TEM	1	9.27	6.17	0.67
180487-86	TEM	1	12.26	12	0.98
180487-86	TEM	1	7.54	7.36	0.98
180487-86	TEM	1	7.07	6.53	0.92
180487-86	TEM	1	8.09	6.38	0.79
180487-86	TEM	1	5.9	5.58	0.95
180487-86	TEM	2	15.15	14.91	0.98
180487-86	TEM	2	8.6	8.06	0.94
180487-86	TEM	2	10.88	10.19	0.94
180487-86	TEM	2	11.58	11.23	0.97
180487-86	TEM	2	8.6	7.55	0.88
180487-86	TEM	2	12.29	12.11	0.99
180487-86	TEM	2	8.77	7.07	0.81
180487-86	TEM	6	8.86	8.57	0.97
180487-86	TEM	6	7.42	6.7	0.90
180487-86	TEM	6	6.91	6.43	0.93
180487-86	TEM	6	5.05	4.77	0.94
180487-86	TEM	6	9.06	7.38	0.81
180487-86	TEM	8	11.25	10.96	0.97
180487-86	TEM	8	14.19	13.68	0.96
180487-86	TEM	8	10.68	10.15	0.95
180487-86	TEM	8	14.5	12.74	0.88
180487-86	TEM	8	17.51	16.07	0.92
180487-86	TEM	8	15.41	15.08	0.98
180487-86	TEM	8	5.2	5.03	0.97
180487-100	TEM	1	20.5	20.25	0.99
180487-100	TEM	1	15.5	15.2	0.98
180487-100	TEM	1	19.5	18.25	0.94
180487-100	TEM	1	16.51	16.27	0.99
180487-100	TEM	1	8.02	7.5	0.94
180487-100	TEM	1	18.5	17.76	0.96
180487-100	TEM	1	10.75	10.5	0.98
180487-100	TEM	1	20.75	19.01	0.92
180487-100	TEM	2	21.55	21.53	1.00

180487-100	TEM	2	28.74	28.42	0.99
180487-100	TEM	2	31.13	29.38	0.94
180487-100	STEM	1	64.91	60.44	0.93
180487-100	STEM	1	65.78	56.6	0.86
180487-100	STEM	1	58.67	53.34	0.91
180487-100	STEM	1	41.79	37.34	0.89
180487-100	STEM	1	52.45	48.89	0.93
180487-100	STEM	1	52.44	51.56	0.98
180487-100	STEM	5	57.92	50.38	0.87
180487-100	STEM	5	56.25	52.83	0.94
180487-100	STEM	5	18.45	18.52	1.00
180487-100	STEM	7	9.83	7.84	0.80
180487-100	STEM	7	8.67	8.17	0.94
180487-100	STEM	9	9.23	8.92	0.97
180487-100	STEM	9	9.69	9.07	0.94
180487-100	STEM	9	8.27	7.25	0.88
180487-100	STEM	9	8.3	7.84	0.94
180487-100	STEM	9	14.63	6.92	0.47
180487-100	STEM	9	1.57	1.55	0.99
180487-100	STEM	9	1.85	1.79	0.97
180487-100	STEM	9	1.52	1.42	0.93

Table S2. List of samples analysed in this study. Route details for operator cabin data is presented in Table S3. Dust concentrations are in (mg/m3) for a 8-hour time-weighted average (TWA).

Sample Name	Filter	Dust conc	Station	Locality
180487-100	PM4	0.93	Piccadilly	Bakerloo line N/B
180487-102	PM4	1.15	Piccadilly	Piccadilly line W/B
180487-107	PM4	0.87	Vauxhall	Northern line S/B
180487-110	PM4	0.82	Tottenham Court Road	Central line W/B
180487-111	PM4	0.7	Tottenham Court Road	Northern line S/B
180487-112	PM4	0.69	Tottenham Court Road	Northern line N/B
180487-12	PM4	-	-	-
180487-28	PM4	-	-	-
180487-49	PM4	0.58	Aldgate East	District Line E/B Platform 2
180487-55	PM4	0.89	Baker Street	Jubilee Line Platform 10 W/B
180487-56	PM4	1.03	Baker Street	Jubilee Line Platform 7 S/B
180487-58	PM4	1.26	Baker Street	Bakerloo Line Platform 9 N/B
180487-70	PM4	1.06	Hampstead Station	Northern line N/B
180487-76	PM4	0.83	King's Cross	Piccadilly line E/B
180487-78	PM4	0.65	King's Cross	Northern line N/B
180487-84	PM4	0.59	Oxford Circus	Personal- LUL Staff (Main ticket hall)
180487-85	PM4	0.78	Oxford Circus	Ticket Hall
180487-86	PM4	1.27	Oxford Circus	Central line E/B
180487-87	PM4	1.07	Oxford Circus	Central line W/B
180487-92	PM4	1.28	Paddington	Bakerloo Line S/B Platform 4
180487-94	PM4	0.67	Paddington	Gate line to Bakerloo line
180487-96	PM4	0.34	Paddington	Gate line to District Line
202073-477	PM2.5	0.33	-	Victoria line
202073-478	PM10	0.2	-	Victoria line
202073-487	PM2.5	0.24	-	Bakerloo line
202073-488	PM10	0.69	-	Bakerloo line
202073-492	PM10	0.54	-	Bakerloo line
202073-495	PM2.5	0.63	-	Bakerloo line
202073-497	PM2.5	0.33	-	Central line
202073-498	PM10	0.33	-	Central line
202073-502	PM10	2.11*	-	Central line
202073-507	PM2.5	0.04	-	Northern line
202073-508	PM10	0.09*	-	Northern line
202073-511	PM2.5	0.31	-	Northern line
202073-512	PM10	1	-	Northern line
202073-514	PM10	0.3	-	Northern line
202073-515	PM2.5	0.91	-	Northern line
202073-537	PM2.5	3.08	-	Piccadilly line
202073-538	PM10	4.17	-	Piccadilly line

Table S3. Route details and timings of sampling PM in the operator cabins.

Sample	Sample type	Date	Start time	Breaks in sampling		Finish time	Route line
				Time off	Time on		
477	PM2.5	22/04/2021	16:25	-	-	20:27	Victoria line
478	PM10	22/04/2021	16:25	-	-	20:27	Victoria line
487	PM2.5	28/04/2021	09:11	10:41	13:05	16:49	Bakerloo line
488	PM10	28/04/2021	09:11	10:41	13:05	16:49	Bakerloo line
492	PM10	29/04/2021	10:39	14:06	15:20	15:54	Bakerloo line
495	PM10	30/04/2021	10:43	-	-	14:04	Bakerloo line
497	PM2.5	12/05/2021	15:44	16:57	18:21	21:27	Central line
498	PM10	12/05/2021	15:44	16:57	18:21	21:27	Central line
502	PM10	13/05/2021	14:15	17:42	19:23	21:15	Central line
507	PM2.5	19/05/2021	14:15	16:36	17:25	21:01	Northern line
508	PM10	19/05/2021	14:15	16:36	17:25	21:01	Northern line
511	PM2.5	20/05/2021	14:16	16:37	17:25	20:36	Northern line
512	PM10	20/05/2021	14:16	16:37	17:25	20:36	Northern line
514	PM2.5	21/05/2021	14:27	17:05	18:27	22:11	Northern line
515	PM10	21/05/2021	14:27	17:05	18:27	22:11	Northern line
537	PM2.5	30/06/2021	08:11	09:14	10:26	12:24	Piccadilly line
538	PM10	30/06/2021	08:11	09:14	10:26	12:24	Piccadilly line

SI References

1. Lagroix, F. & Guyodo, Y. A new tool for separating the magnetic mineralogy of complex mineral assemblages from low temperature magnetic behavior. *Front Earth Sci (Lausanne)* **5**, (2017).

2. Chambolle, A. & Pock, T. A first-order primal-dual algorithm for convex problems with applications to imaging. *J Math Imaging Vis* **40**, (2011).

3. Gao, X. *et al.* New High-Temperature Dependence of Magnetic Susceptibility-Based Climofunction for Quantifying Paleoprecipitation From Chinese Loess. *Geochemistry, Geophysics, Geosystems* **20**, (2019).

4. Maher, B. A. Magnetic properties of some synthetic sub-micron magnetites. *Geophysical Journal* **94**, (1988).

5. Thompson, R. & Oldfield, F. *Environmental Magnetism*. *Environmental Magnetism* (1986). doi:10.1007/978-94-011-8036-8.

6. Harrison, R. J. *et al.* An Improved Algorithm for Unmixing First-Order Reversal Curve Diagrams Using Principal Component Analysis. *Geochemistry, Geophysics, Geosystems* **19**, (2018).

7. Zhao, X., Heslop, D. & Roberts, A. P. A protocol for variable-resolution first-order reversal curve measurements. *Geochemistry, Geophysics, Geosystems* **16**, (2015).

8. Zhao, X. *et al.* Magnetic domain state diagnosis using hysteresis reversal curves. *J Geophys Res Solid Earth* **122**, (2017).

9. Roberts, A. P. *et al.* Unlocking information about fine magnetic particle assemblages from first-order reversal curve diagrams: Recent advances. *Earth-Science Reviews* vol. 227 Preprint at https://doi.org/10.1016/j.earscirev.2022.103950 (2022).

10. Harrison, R. J. *et al.* Simulation of Remanent, Transient, and Induced FORC Diagrams for Interacting Particles With Uniaxial, Cubic, and Hexagonal Anisotropy. *J Geophys Res Solid Earth* **124**, (2019).

11. Tung, P.-Y., Sheikh, H. A., Ball, M. R., Nabiei, F. & Harrison, R. SIGMA: Spectral Interpretation using Gaussian Mixtures and Autoencoder. *Earth and Space Science Open Archive* 22 (2022) doi:10.1002/essoar.10511396.1.
